# Is It the Right Time for Routine Clinical Application of Stem Cells in Reproductive Medicine?

**Published:** 2018

**Authors:** Mohammad Reza Sadeghi

Infertility as a universal public health topic affects about 15% of couples in reproductive age of 18–45 worldwide. According to epidemiologic studies, it is steadily increasing in the industrialized countries due to envirnomental factors and habitual changes in human's lifestyle. WHO estimates that about 60–80 million of infertile couples require medical intervention to get pregnant every year. In spite of all the progress and development in the diagnosis and treatment of infertility, assissted reproductive technologies (ARTs) are able to help only less than 70% of them during the first 5 years of their attempt through assissted concieving using either their own gametes or the reproductive capacity of the third party (1).

Despite the advances in using gametes and embryo donation for infertility treatment, a relatively large number of couples are unable to accept third party conception as the final option for their treatment. They request for gametes production from their own geneome through advances in stem cell biology and regenerative medicine. However, the exaggerating of media about priliminary results of scientific research is more than reality which tiggers the premature request of infertile couples.

Stem cell biology and regenerative medicine is a new field of biomedicine and is going through its initial and introductory stages. The first human embryonic stem cells were created by the team of James Thomson, an American developmental biologist, in 1998. Stem cells are characterized as undifferentiated cells with the ability of proliferation, self-renewal, and differentiation almost into any of the over 200 cell types in the body. The stem cells application in translational research leads to enhancement and promotion in the field of regenerative medicine. Currently, stem cells provide massive promise for cell therapy, tissue engineering, and regenerative medicine as well as using for pharmaceutical and biotechnological purposes (2).

The past decade developments were accompanied with extraordinary growth of experimental therapies of regenerative medicine, presented in clinical practice. The outcomes vary from clear positive clinical effects for a few irremediable diseases to relatively less or no effects for others. The causes of these inconsistent consequences are in the veil of ambiguity. Furthermore, experimental cell therapies on patients are more expensive. Although rich goverments and international companies financed these experimental therapies, there is no guarantee for returns of their investments. Therefore, the success of limited number of these therapies leads to more expensive treatment procedures that circumscribe their routine application for everyone at the beginning. In addition, these experimental therapies may expose the patients to unknown risks which are not predictable (3).

Several experimental therapies of stem cell were performed in reproductive medicine with the aim of artifical gametes generation, endometrial reconstruction, erectile dysfunction restoration, and vaginal amendment. The unproven, untested and often unsafe stem cells treatments are ferquent in the field of reproductive biomedicine. Up to now, all of stem cell-based treatments in reproductive medicine are limited to clinical trials. Particularly, the stem cell based clinical experiments on *in vitro* or de novo generation of gametes failed to improve reproductive function of infertile cases (4).

Stem cell-based technologies similar to large numbers of other technologies have been introduced and prematurely presented to infertile couples over the last 30 years and this process is not going to stop. However, before transferring a technology to reproductive medicine, its evaluation in terms of effectiveness, safety and cost effectiveness should be completed. Moreover, the appeal for introduction and using new technologies by the community is obvious regarding our lack of knowledge about infertility causes and its low success rate. It should be noted that we are all looking for advances in the field of ART and wish to provide the best possible treatment plan for our patients. But we must always look for the best practice in our clinics (5).

Today, often public media publish incomplete early results of scientific research and report in congruent demands in the community. Therefore, patients often ask the physicians for their favorite choices and media propagates some issues or even some research results from websites which may be fake. In fact, financial profit, advertising of pharmaceutical and instrument companies or even emotional pressures of patients should not lead us to wrong directions. The precipitant in using new technology without validation through well designed numerouse clinical trials undoubtedly would be the reason for wasting the time and financial resources of patients and health systems, and may even exert irreparable damage on patient health. Therefore, with regard to the above mentioned facts, it is essential to prevent routine and broad application of stem cells in the treatment of infertility, and we need to pay more attention in the procedure of designing clinical trials for generalizability of the results. Nevertheless however, we believe that stem cells and regenerative medicine will play a significant and critical role in the future of medicine and human health.
